# Supplementary figures and images for: Analysis of co-isogenic prion protein deficient mice reveals behavioral deficits, learning impairment, and enhanced hippocampal excitability
Source: BMC Biol. 2022 Jan 13;20:17. doi: 10.1186/s12915-021-01203-0 (PMC8759182; doi:10.1186/s12915-021-01203-0)

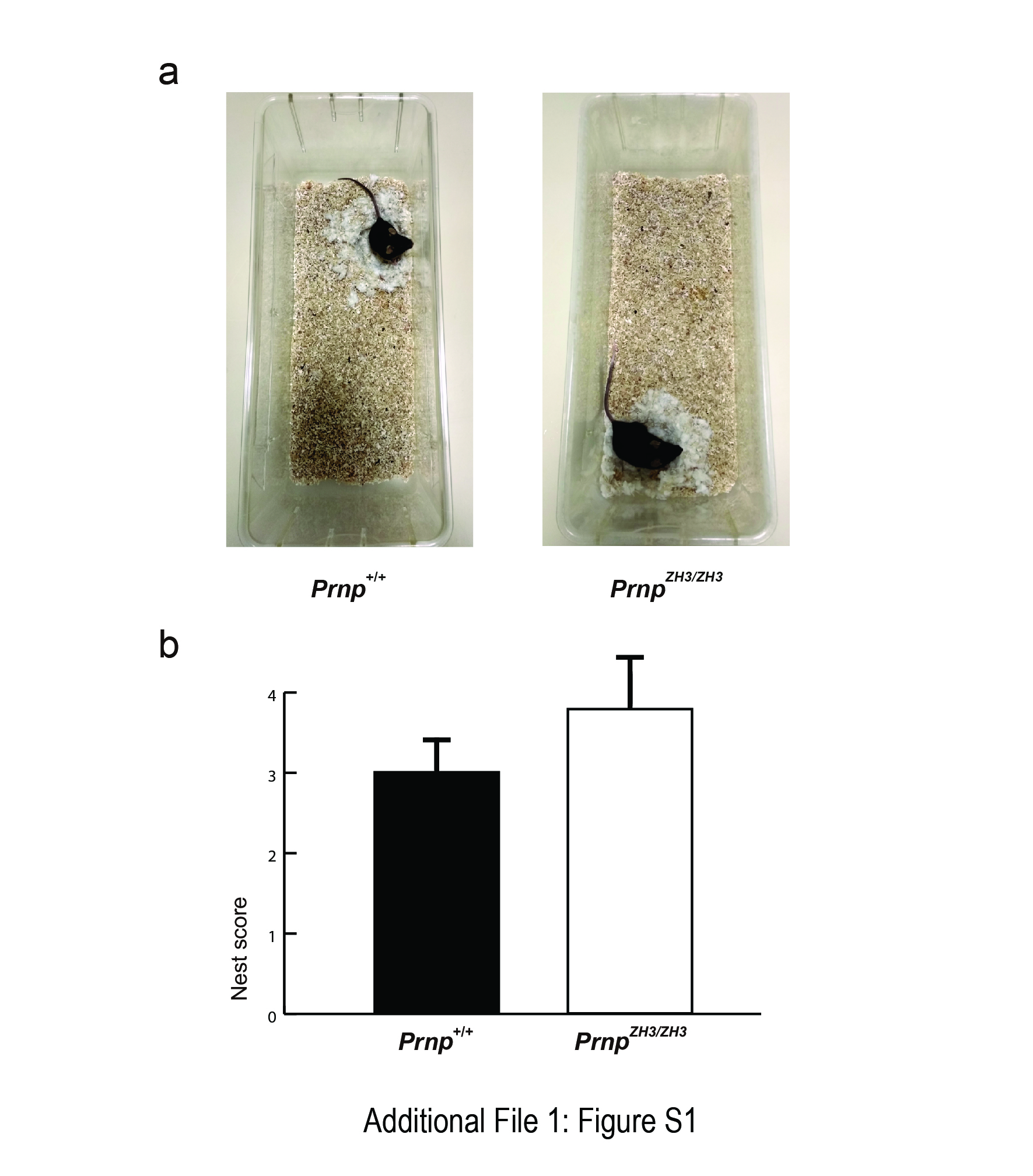

Supplement: Supplementary file 1 — Additional file 1: Figure S1. PrnpZH3/ZH3 mice showed similar nest-building behavior to wild-type mice. a, Representative images of nests constructed by Prnp+/+ (left) and PrnpZH3/ZH3 mice (right). b, Quantification illustrating the mean of the nest score (see Material and Methods for details). Data are presented as mean ± S.E.M. Source data and individual data values are available in Additional file 10. [file 12915_2021_1203_MOESM1_ESM.jpg]

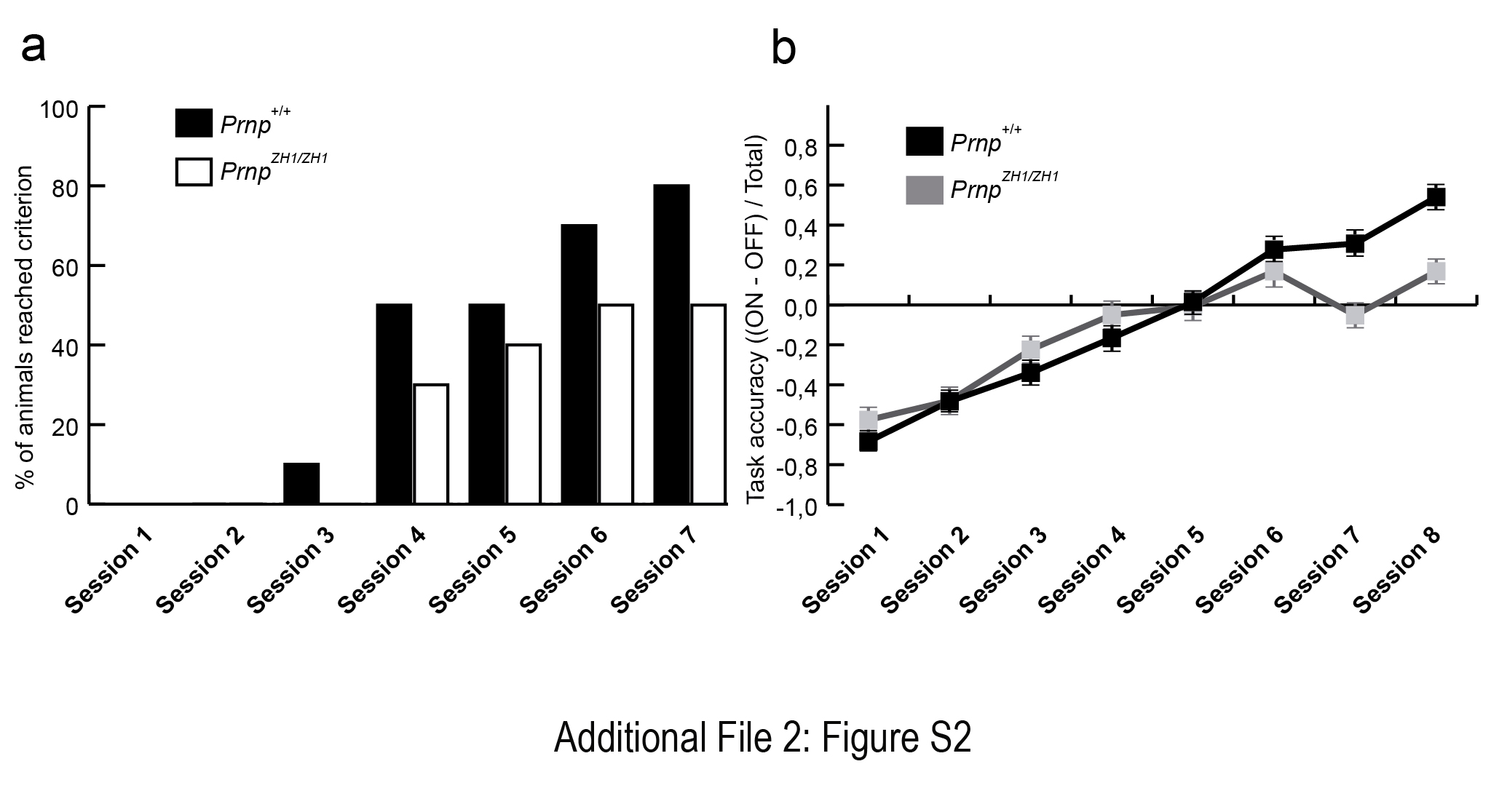

Supplement: Supplementary file 2 — Additional file 2: Figure S2. PrnpZH1/ZH1 mice failed to acquire an instrumental learning test in the Skinner box test using a fixed ratio (1:1) schedule. a, Percentage of Prnp+/+ and PrnpZH1/ZH1 mice reached the selected criterion during the training sessions. b, Task accuracy ((lever presses during light ON – lever presses during the light OFF) / Total number of lever presses) during the light ON / light OFF conditioning paradigm. Data are presented as a percentage in a and as mean ± S.E.M. in b. Source data and individual data values are available in Additional file 10. [file 12915_2021_1203_MOESM2_ESM.jpg]

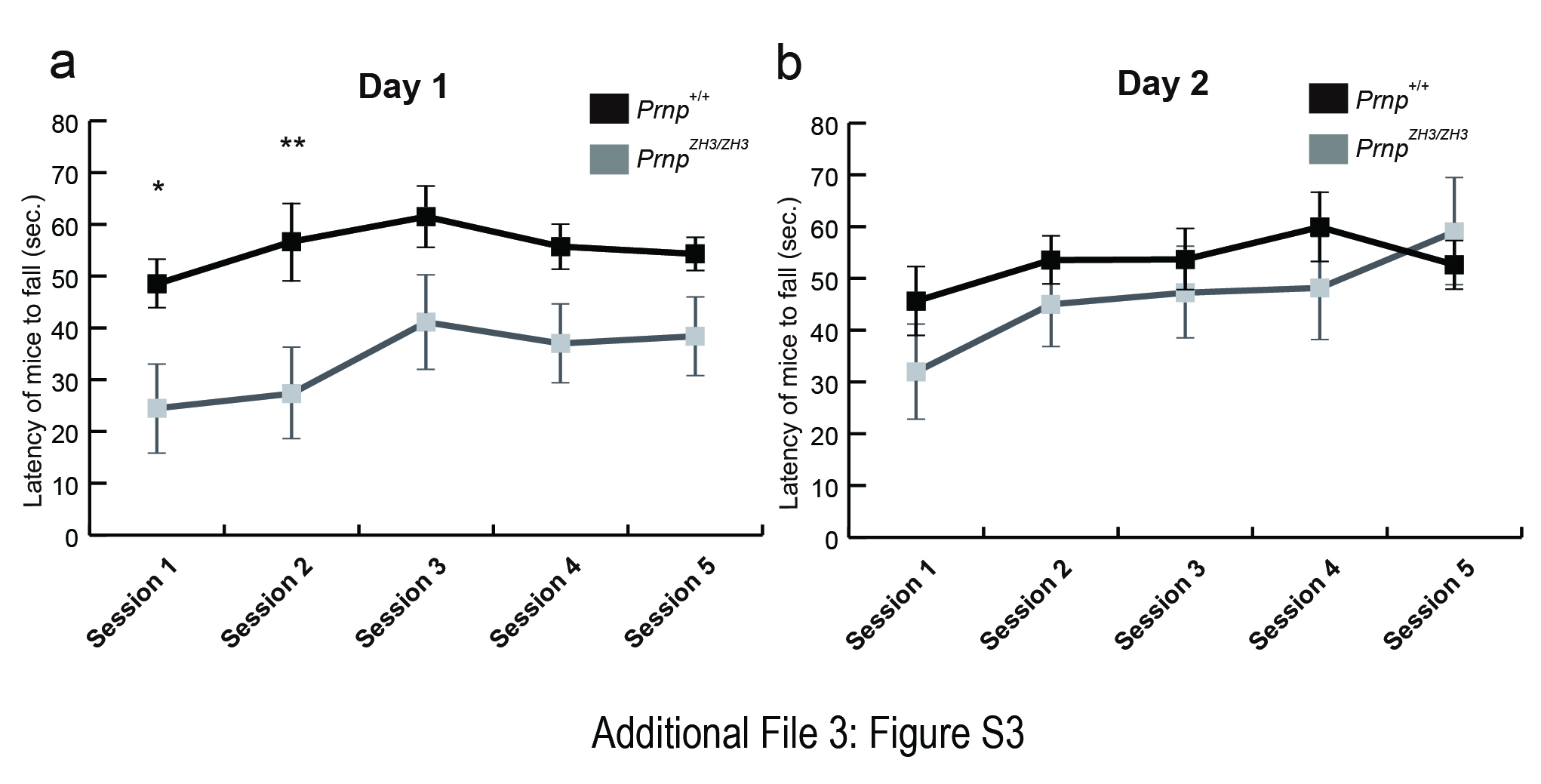

Supplement: Supplementary file 3 — Additional file 3: Figure S3. PrnpZH3/ZH3 mice have deficits in acquiring instrumental learning but not motor impairment in the Rotarod test. After a training session in the Rotarod, mice were tested two consecutive days with 5 sessions in each. Latency of mice to fall (sec.) from the Rotarod in the first day (a) or in the second (b). Data are presented as mean ± S.E.M.. *p < 0.05 and **p < 0.01, two-way ANOVA + Bonferroni’s multiple comparisons test. Source data and individual data values are available in Additional file 10. [file 12915_2021_1203_MOESM3_ESM.jpg]

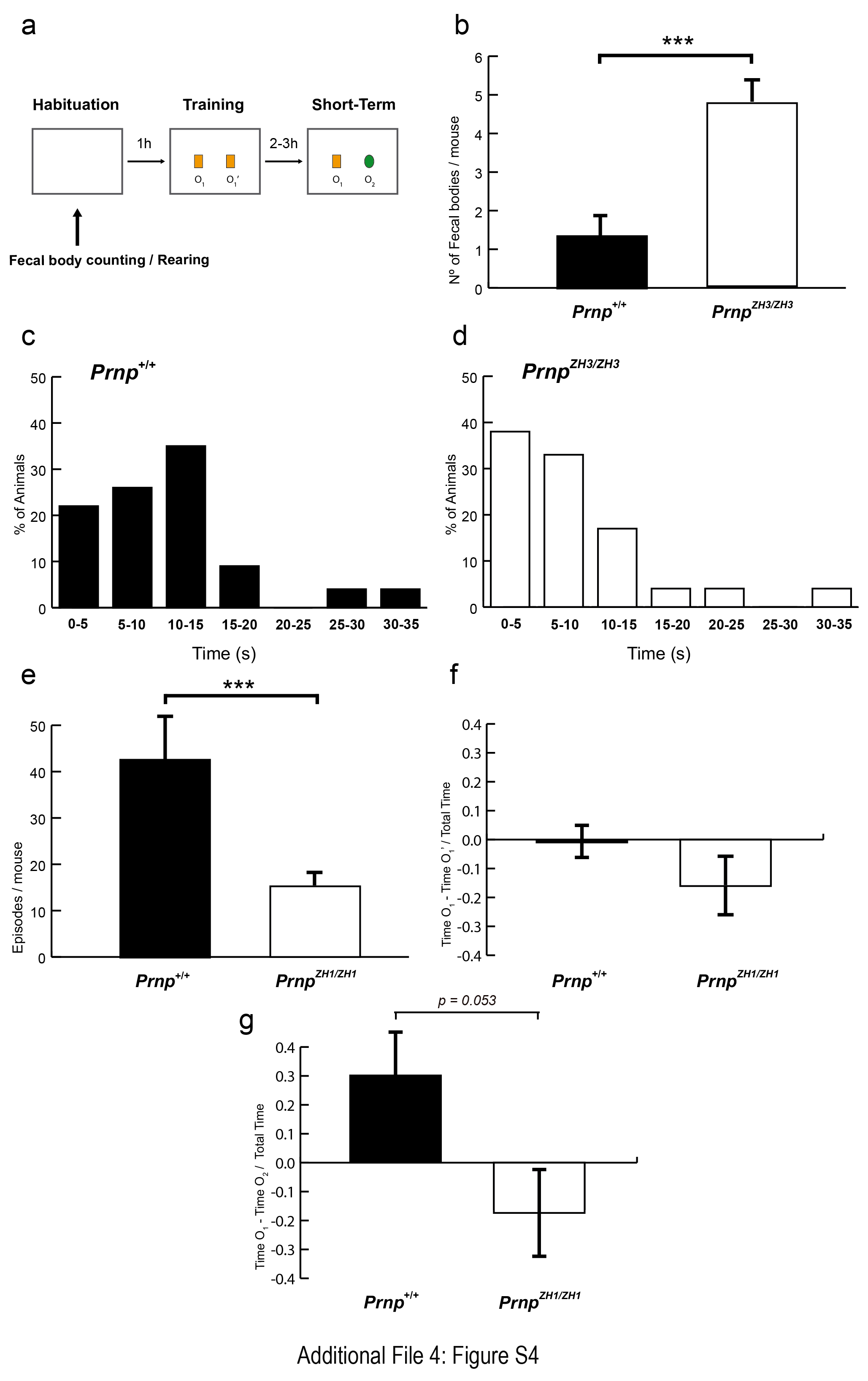

Supplement: Supplementary file 4 — Additional file 4: Figure S4. Stressed-like behavior in PrnpZH3/ZH3 mice impairing object recognition test performance. PrnpZH1/ZH1 mice also showed stressed-like behavior and failed to acquire short-term memory. a, Scheme of object recognition protocol that consisted of 3 sessions (10 min each). First, mice were habituated in the empty arena. One hour later, they were placed again in the arena with two identical objects for the training session. Finally, 2-3 h later, they were once more placed in the arena, replacing one object with a novel one for the short-term memory test. Fecal bodies (PrnpZH3/ZH3) and rearing episodes (PrnpZH1/ZH1) were counted in the habituation session as an indicator of animal stress. b, Number of fecal bodies generated per animal (Prnp+/+ and PrnpZH3/ZH3) during the habituation session. Data are presented as mean ± S.E.M. c-d Time that Prnp+/+ (c), and PrnpZH3/ZH3 (d) mice interacted with the objects in the short-term test. Data are presented as the percentage of mice interacting with the object in each time interval (0 to 35 s). e, Number of rearing episodes in the habituation session (Prnp+/+ and PrnpZH1/ZH1). f-g, Relative time that the two exposed objects are explored to the total time invested. Object 1 (O1) and object 1’ (O1’) in the training session (f) and number, and object 1 (O1) and object 2 (O2) in the short-term memory test (g). Data are presented as percentages in C-D and as mean ± S.E.M. in b, e, f, g. *p < 0.05 and ***p < 0.001, Mann-Whitney U non-parametric test. Source data and individual data values are available in Additional file 10. [file 12915_2021_1203_MOESM4_ESM.jpg]

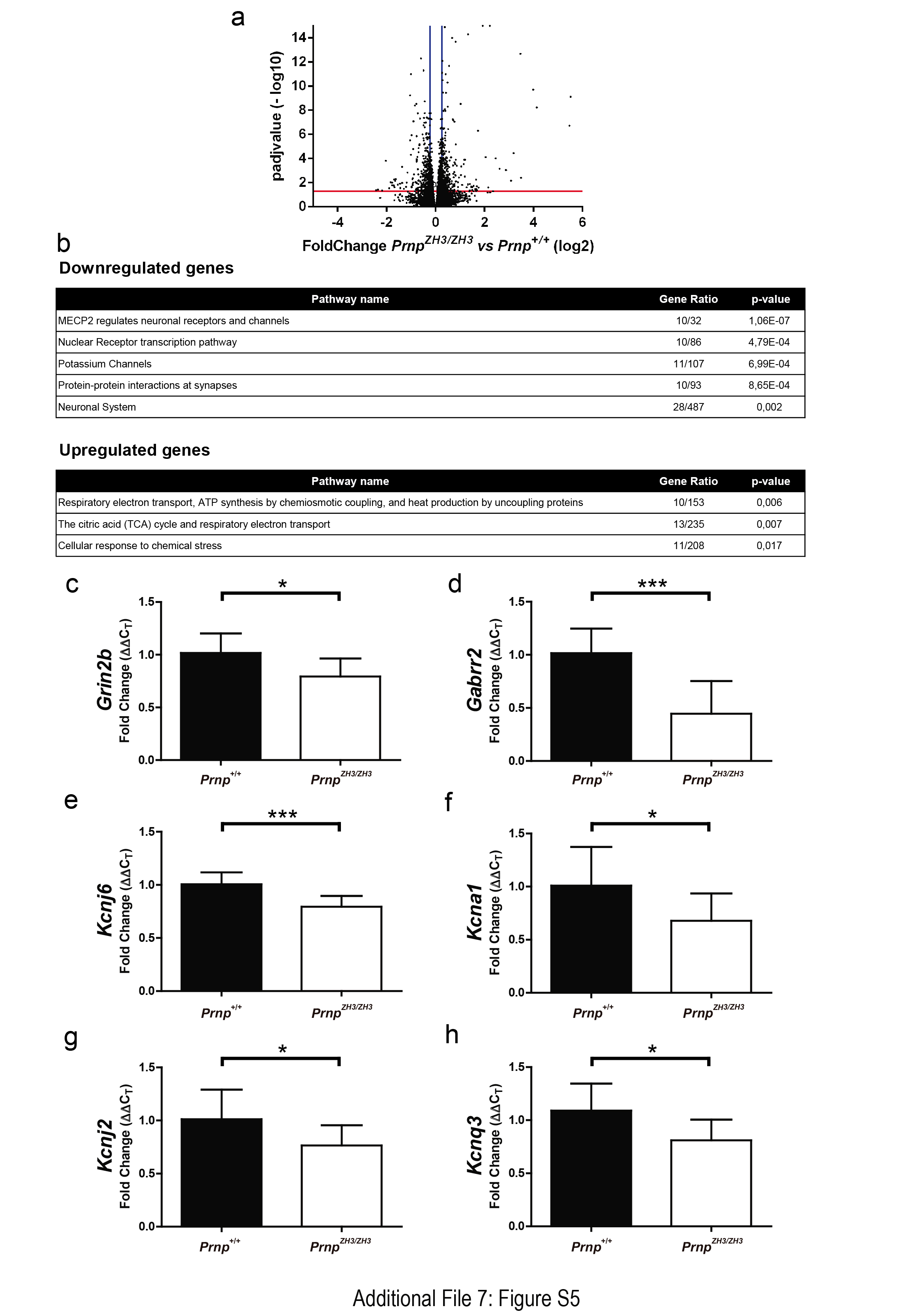

Supplement: Supplementary file 7 — Additional file 7: Figure S5. Gene ontology of the genes significantly downregulated and upregulated in PrnpZH3/ZH3 hippocampus compared to Prnp+/+. a, Volcano plot of the protein-coding genes analyzed in the RNAseq (~ 16.000). In the X-axis is plotted the Fold change (log2) and in the Y-axis the padj (-log10). The red line separates the significantly expressed genes (padj < 0,05). The blue lines indicate the 0,85 and 1,2 fold changes. b, Gene ontology analysis of the downregulated and upregulated genes with the Reactome software (see Materials and Methods for details). Validation of the main genes altered by RT-qPCR: Grin2b (c), Gabrr2 (d), Kcnj6 (e), Kcna1 (f), Kcnj2 (g), and Kcnq3 (h). Data are presented as mean ± S.E.M. *p < 0.05 and ***p < 0.001. Source data and individual data values are available in Additional file 10. [file 12915_2021_1203_MOESM7_ESM.jpg]

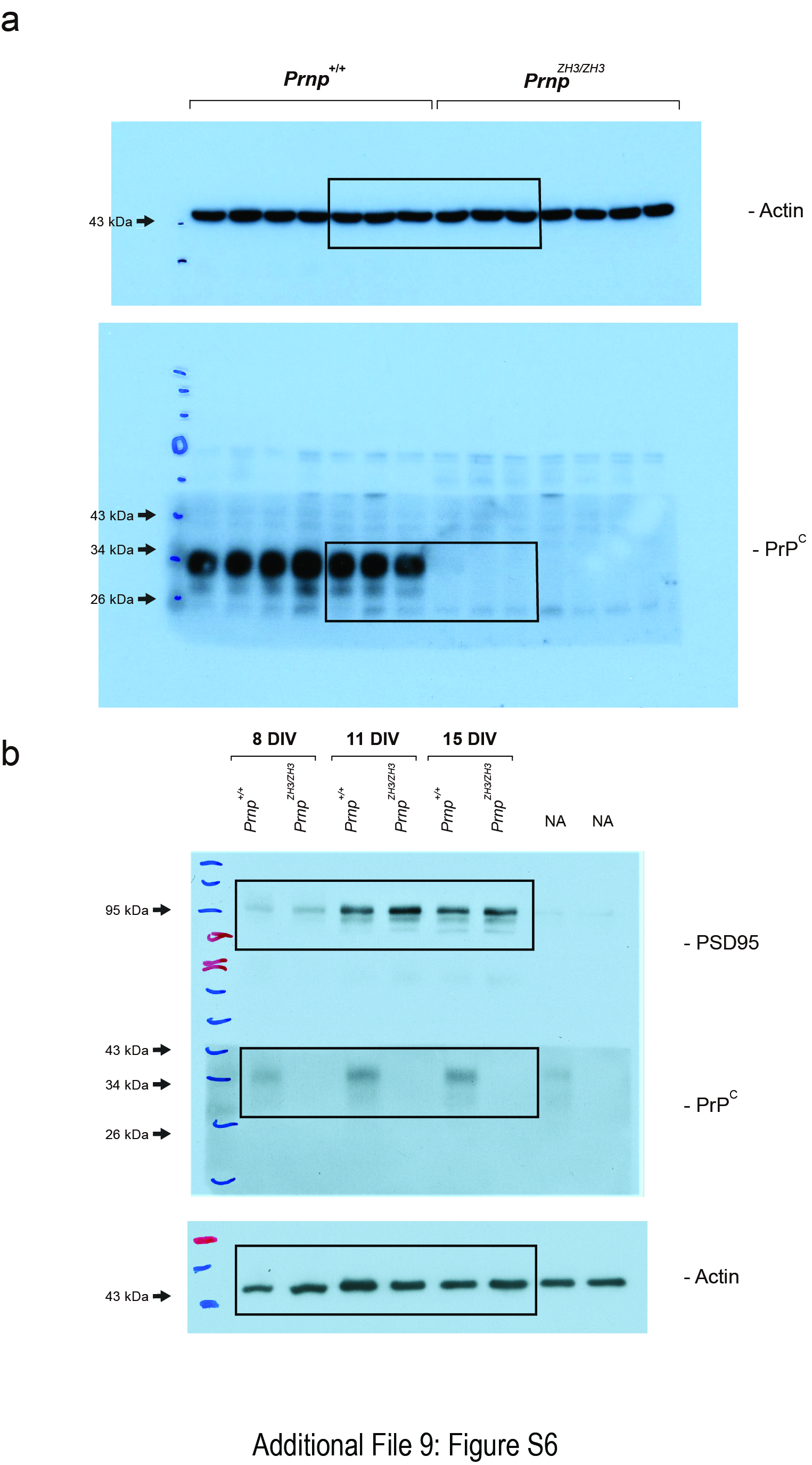

Supplement: Supplementary file 9 — Additional file 9: Figure S6. Images of the original, uncropped blots for Figs. 1A and 6A. [file 12915_2021_1203_MOESM9_ESM.jpg]
