# Supplementary material for: Analysis of co-isogenic prion protein deficient mice reveals behavioral deficits, learning impairment, and enhanced hippocampal excitability
Source: BMC Biol. 2022 Jan 13;20:17. doi: 10.1186/s12915-021-01203-0 (PMC8759182; doi:10.1186/s12915-021-01203-0)
Supplement: Supplementary file 5 — Additional file 5: Table S1. List of the protein-coding significantly downregulated genes in PrnpZH3/ZH3 hippocampus compared to Prnp+/+. [file 12915_2021_1203_MOESM5_ESM.pdf]

Additional File 5: Table S1

Downregulated genes in *Prnp* $^{ZH3/ZH3}$ compared to *Prnp* $^{+/+}$

Gene	Name	padj_Ko vs Wt
**Abcb1b**	ATP-binding cassette, sub-family B (MDR/TAP), member 1B(Abcb1b)	3,13E-03
**Actr2**	ARP2 actin-related protein 2(Actr2)	2,71E-04
**Adcy9**	adenylate cyclase 9(Adcy9)	4,55E-04
**Adgrl3**	adhesion G protein-coupled receptor L3(Adgrl3)	5,00E-04
**Afap1l1**	actin filament associated protein 1-like 1(Afap1l1)	1,51E-03
**Aff1**	AF4/FMR2 family, member 1(Aff1)	4,40E-02
**Ago3**	argonaute RISC catalytic subunit 3(Ago3)	3,64E-02
**AI593442**	expressed sequence AI593442(AI593442)	9,02E-03
**Aim2**	absent in melanoma 2(Aim2)	1,49E-03
**Aldh1l2**	aldehyde dehydrogenase 1 family, member L2(Aldh1l2)	1,61E-04
**Aldh7a1**	aldehyde dehydrogenase family 7, member A1(Aldh7a1)	1,02E-04
**Ang**	angiogenin, ribonuclease, RNase A family, 5(Ang)	1,07E-02
**Ank3**	ankyrin 3, epithelial(Ank3)	7,57E-03
**Anln**	anillin, actin binding protein(Anln)	4,66E-02
**Ano1**	anoctamin 1, calcium activated chloride channel(Ano1)	2,45E-02
**Aoc1**	amine oxidase, copper-containing 1(Aoc1)	1,61E-02
**Ap4b1**	adaptor-related protein complex AP-4, beta 1(Ap4b1)	2,54E-02
**Arfgef3**	ARFGEF family member 3(Arfgef3)	1,28E-02
**Arhgap27**	Rho GTPase activating protein 27(Arhgap27)	2,37E-02
**Arhgef19**	Rho guanine nucleotide exchange factor (GEF) 19(Arhgef19)	2,25E-02
**Arhgef37**	Rho guanine nucleotide exchange factor (GEF) 37(Arhgef37)	2,36E-02
**Arsi**	arylsulfatase i(Arsi)	3,15E-02
**Arsj**	arylsulfatase J(Arsj)	1,99E-06
**Atf4**	activating transcription factor 4(Atf4)	1,85E-06
**Atf5**	activating transcription factor 5(Atf5)	5,06E-04
**Atm**	ataxia telangiectasia mutated(Atm)	5,52E-03
**Atp2b1**	ATPase, Ca++ transporting, plasma membrane 1(Atp2b1)	9,92E-04
**Atp5g1**	ATP synthase, H+ transporting, mitochondrial F0 complex, subunit C1 (subunit 9)(Atp5g1)	1,08E-07
**Atrx**	alpha thalassemia/mental retardation syndrome X-linked(Atrx)	1,41E-03
**Bach2**	BTB and CNC homology, basic leucine zipper transcription factor 2(Bach2)	5,75E-03
**Bbs12**	Bardet-Biedl syndrome 12 (human)(Bbs12)	2,10E-05
**Bclaf1**	BCL2-associated transcription factor 1(Bclaf1)	3,73E-05
**Bdnf**	brain derived neurotrophic factor(Bdnf)	1,01E-07
**Best3**	bestrophin 3(Best3)	3,27E-03
**Bex1**	brain expressed X-linked 1(Bex1)	4,41E-04
**Bhlhe41**	basic helix-loop-helix family, member e41(Bhlhe41)	6,25E-04
**Birc6**	baculoviral IAP repeat-containing 6(Birc6)	2,16E-02
**Birc7**	baculoviral IAP repeat-containing 7 (livin)(Birc7)	3,64E-02
**Bmpr2**	bone morphogenetic protein receptor, type II (serine/threonine kinase)(Bmpr2)	9,38E-03
**Bok**	BCL2-related ovarian killer(Bok)	5,93E-03
**Brwd3**	bromodomain and WD repeat domain containing 3(Brwd3)	2,55E-02
**Btaf1**	B-TFIID TATA-box binding protein associated factor 1(Btaf1)	7,67E-06
**C1ql2**	complement component 1, q subcomponent-like 2(C1ql2)	4,41E-02
**C2cd4a**	C2 calcium-dependent domain containing 4A(C2cd4a)	2,62E-02
**Cacna1e**	calcium channel, voltage-dependent, R type, alpha 1E subunit(Cacna1e)	2,13E-02
**Cacnb4**	calcium channel, voltage-dependent, beta 4 subunit(Cacnb4)	1,07E-02
**Cacng5**	calcium channel, voltage-dependent, gamma subunit 5(Cacng5)	2,19E-02
**Cacul1**	CDK2 associated, cullin domain 1(Cacul1)	2,55E-03
**Camp**	cathelicidin antimicrobial peptide(Camp)	4,17E-02
**Caps2**	calcyphosphine 2(Caps2)	8,80E-03
**Capza1**	capping protein (actin filament) muscle Z-line, alpha 1(Capza1)	1,41E-03
**Car9**	carbonic anhydrase 9(Car9)	2,05E-03
**Card6**	caspase recruitment domain family, member 6(Card6)	1,29E-02
**Cars**	cysteinyl-tRNA synthetase(Cars)	3,87E-08
**Cast**	calpastatin(Cast)	4,76E-02
**Cdh26**	cadherin-like 26(Cdh26)	1,54E-02
**Cdh8**	cadherin 8(Cdh8)	3,50E-03
**Cdhr4**	cadherin-related family member 4(Cdhr4)	1,37E-02
**Cfap157**	cilia and flagella associated protein 157(Cfap157)	4,37E-02
**Cgn**	cingulin(Cgn)	4,14E-02
**Chac1**	ChaC, cation transport regulator 1(Chac1)	1,37E-06
**Chaf1a**	chromatin assembly factor 1, subunit A (p150)(Chaf1a)	4,67E-02
**Chl1**	cell adhesion molecule L1-like(Chl1)	1,80E-04
**Chrna1**	cholinergic receptor, nicotinic, alpha polypeptide 1 (muscle)(Chrna1)	4,38E-02
**Chst9**	carbohydrate (N-acetylgalactosamine 4-0) sulfotransferase 9(Chst9)	2,01E-04
**Ciart**	circadian associated repressor of transcription(Ciart)	1,13E-02
**Clstn2**	calsyntenin 2(Clstn2)	1,72E-04
**Cntn3**	contactin 3(Cntn3)	4,66E-03
**Cort**	cortistatin(Cort)	1,80E-04
**Cpeb4**	cytoplasmic polyadenylation element binding protein 4(Cpeb4)	1,96E-03
**Crhbp**	corticotropin releasing hormone binding protein(Crhbp)	9,83E-03
**Csmd3**	CUB and Sushi multiple domains 3(Csmd3)	3,82E-02
**Cxcl5**	chemokine (C-X-C motif) ligand 5(Cxcl5)	7,17E-03
**D130040H23Rik**	RIKEN cDNA D130040H23 gene(D130040H23Rik)	4,62E-02

Additional File 5: Table S1

**Dap3**	death associated protein 3(Dap3)	3,10E-04
**Dbf4**	DBF4 zinc finger(Dbf4)	1,82E-02
**Dbh**	dopamine beta hydroxylase(Dbh)	4,18E-02
**Defb42**	defensin beta 42(Defb42)	1,70E-02
**Dennd2c**	DENN/MADD domain containing 2C(Dennd2c)	3,55E-02
**Dgkh**	diacylglycerol kinase, eta(Dgkh)	2,54E-04
**Dgki**	diacylglycerol kinase, iota(Dgki)	1,09E-03
**Dhx33**	DEAH (Asp-Glu-Ala-His) box polypeptide 33(Dhx33)	1,16E-04
**Dnah9**	dynein, axonemal, heavy chain 9(Dnah9)	8,65E-04
**Dnajb14**	DnaJ heat shock protein family (Hsp40) member B14(Dnajb14)	8,00E-03
**Dock10**	dedicator of cytokinesis 10(Dock10)	3,85E-02
**Dock4**	dedicator of cytokinesis 4(Dock4)	1,90E-03
**Drd5**	dopamine receptor D5(Drd5)	2,19E-02
**Drosha**	drosha, ribonuclease type III(Drosha)	5,56E-03
**Dsel**	dermatan sulfate epimerase-like(Dsel)	5,34E-04
**Dthd1**	death domain containing 1(Dthd1)	9,87E-03
**Duox2**	dual oxidase 2(Duox2)	1,50E-04
**Eda2r**	ectodysplasin A2 receptor(Eda2r)	2,42E-02
**Eno1**	enolase 1, alpha non-neuron(Eno1)	6,22E-03
**Eomes**	eomesodermin(Eomes)	2,45E-02
**Epha6**	Eph receptor A6(Epha6)	1,85E-04
**Epm2aip1**	EPM2A (laforin) interacting protein 1(Epm2aip1)	5,71E-03
**Eps8l1**	EPS8-like 1(Eps8l1)	9,80E-03
**Epx**	eosinophil peroxidase(Epx)	8,84E-03
**Etaa1**	Ewing tumor-associated antigen 1(Etaa1)	4,94E-03
**Exosc9**	exosome component 9(Exosc9)	2,80E-04
**Farp1**	FERM, RhoGEF (Arhgef) and pleckstrin domain protein 1 (chondrocyte-derived)(Farp1)	4,75E-04
**Fgfbp3**	fibroblast growth factor binding protein 3(Fgfbp3)	1,31E-04
**Filip1**	filamin A interacting protein 1(Filip1)	3,22E-03
**Fkbp10**	FK506 binding protein 10(Fkbp10)	9,60E-04
**Fnip1**	folliculin interacting protein 1(Fnip1)	2,16E-04
**Foxk1**	forkhead box K1(Foxk1)	9,17E-05
**Frmpd2**	FERM and PDZ domain containing 2(Frmpd2)	9,24E-03
**Frrs1l**	ferric-chelate reductase 1 like(Frrs1l)	4,36E-04
**Fry**	FRY microtubule binding protein(Fry)	1,39E-04
**Fstl4**	follistatin-like 4(Fstl4)	3,43E-04
**Fzd3**	frizzled class receptor 3(Fzd3)	3,44E-02
**Gabrr2**	gamma-aminobutyric acid (GABA) C receptor, subunit rho 2(Gabrr2)	4,91E-03
**Garem1**	GRB2 associated regulator of MAPK1 subtype 1(Garem1)	2,54E-06
**Gbe1**	glucan (1,4-alpha-), branching enzyme 1(Gbe1)	4,88E-02
**Gdf9**	growth differentiation factor 9(Gdf9)	6,70E-03
**Gemin6**	gem (nuclear organelle) associated protein 6(Gemin6)	8,98E-03
**Gfra2**	glial cell line derived neurotrophic factor family receptor alpha 2(Gfra2)	2,69E-02
**Ggps1**	geranylgeranyl diphosphate synthase 1(Ggps1)	1,88E-03
**Gipr**	gastric inhibitory polypeptide receptor(Gipr)	1,15E-06
**Glis3**	GLIS family zinc finger 3(Glis3)	3,45E-02
**Glp2r**	glucagon-like peptide 2 receptor(Glp2r)	2,58E-02
**Gnpda1**	glucosamine-6-phosphate deaminase 1(Gnpda1)	2,98E-05
**Gpc4**	glypican 4(Gpc4)	5,02E-03
**Gpr150**	G protein-coupled receptor 150(Gpr150)	1,62E-02
**Gpr151**	G protein-coupled receptor 151(Gpr151)	2,76E-02
**Gpr21**	G protein-coupled receptor 21(Gpr21)	1,90E-02
**Gpr22**	G protein-coupled receptor 22(Gpr22)	2,77E-02
**Grin2a**	glutamate receptor, ionotropic, NMDA2A (epsilon 1)(Grin2a)	7,81E-03
**Grin2b**	glutamate receptor, ionotropic, NMDA2B (epsilon 2)(Grin2b)	5,96E-05
**Gtpbp10**	GTP-binding protein 10 (putative)(Gtpbp10)	1,11E-04
**Gucy1a2**	guanylate cyclase 1, soluble, alpha 2(Gucy1a2)	2,12E-03
**Gucy2g**	guanylate cyclase 2g(Gucy2g)	3,87E-03
**Hcn1**	hyperpolarization-activated, cyclic nucleotide-gated K+ 1(Hcn1)	1,70E-02
**Hdac1**	histone deacetylase 1(Hdac1)	1,70E-03
**Hectd2**	HECT domain containing 2(Hectd2)	2,05E-04
**Hecw2**	HECT, C2 and WW domain containing E3 ubiquitin protein ligase 2(Hecw2)	4,59E-03
**Henmt1**	HEN1 methyltransferase homolog 1 (Arabidopsis)(Henmt1)	8,18E-03
**Herc2**	HECT and RLD domain containing E3 ubiquitin protein ligase 2(Herc2)	7,55E-03
**Hfe**	hemochromatosis(Hfe)	3,41E-02
**Hfm1**	HFM1, ATP-dependent DNA helicase homolog(Hfm1)	7,18E-05
**Hjurp**	Holliday junction recognition protein(Hjurp)	5,99E-05
**Hk2**	hexokinase 2(Hk2)	7,85E-03
**Hmgn2**	high mobility group nucleosomal binding domain 2(Hmgn2)	2,75E-03
**Hpdl**	4-hydroxyphenylpyruvate dioxygenase-like(Hpdl)	3,81E-02
**Hspb6**	heat shock protein, alpha-crystallin-related, B6(Hspb6)	5,71E-03
**Htr5b**	5-hydroxytryptamine (serotonin) receptor 5B(Htr5b)	1,43E-02
**Hunk**	hormonally upregulated Neu-associated kinase(Hunk)	1,17E-02
**Iars2**	isoleucine-tRNA synthetase 2, mitochondrial(Iars2)	7,82E-06
**Ide**	insulin degrading enzyme(Ide)	3,91E-09
**Idua**	iduronidase, alpha-L-(Idua)	2,12E-03
**Igfbpl1**	insulin-like growth factor binding protein-like 1(Igfbpl1)	4,39E-02
**Igtp**	interferon gamma induced GTPase(Igtp)	3,55E-02
**Il1rap**	interleukin 1 receptor accessory protein(Il1rap)	1,15E-02

Additional File 5: Table S1

**Ildr2**	immunoglobulin-like domain containing receptor 2(Ildr2)	8,67E-03
**Inhba**	inhibin beta-A(Inhba)	4,55E-02
**Ints7**	integrator complex subunit 7(Ints7)	1,81E-09
**Irgm2**	immunity-related GTPase family M member 2(Irgm2)	3,15E-02
**Itpka**	inositol 1,4,5-trisphosphate 3-kinase A(Itpka)	3,91E-02
**Izumo4**	IZUMO family member 4(Izumo4)	4,17E-02
**Kansl1l**	KAT8 regulatory NSL complex subunit 1-like(Kansl1l)	3,30E-03
**Kcna1**	potassium voltage-gated channel, shaker-related subfamily, member 1(Kcna1)	7,51E-07
**Kcna4**	potassium voltage-gated channel, shaker-related subfamily, member 4(Kcna4)	4,41E-02
**Kcnb2**	potassium voltage gated channel, Shab-related subfamily, member 2(Kcnb2)	7,68E-04
**Kcnh5**	potassium voltage-gated channel, subfamily H (eag-related), member 5(Kcnh5)	1,02E-02
**Kcnh7**	potassium voltage-gated channel, subfamily H (eag-related), member 7(Kcnh7)	1,44E-02
**Kcnj2**	potassium inwardly-rectifying channel, subfamily J, member 2(Kcnj2)	3,05E-03
**Kcnj6**	potassium inwardly-rectifying channel, subfamily J, member 6(Kcnj6)	7,00E-05
**Kcnq3**	potassium voltage-gated channel, subfamily Q, member 3(Kcnq3)	5,24E-03
**Kcns1**	K+ voltage-gated channel, subfamily S, 1(Kcns1)	2,25E-02
**Kctd16**	potassium channel tetramerisation domain containing 16(Kctd16)	3,49E-03
**Kctd4**	potassium channel tetramerisation domain containing 4(Kctd4)	2,37E-02
**Khdrbs1**	KH domain containing, RNA binding, signal transduction associated 1(Khdrbs1)	6,55E-06
**Klhl40**	kelch-like 40(Klhl40)	4,26E-02
**Krt12**	keratin 12(Krt12)	4,76E-05
**Krt222**	keratin 222(Krt222)	6,70E-04
**Krt9**	keratin 9(Krt9)	1,76E-08
**Lactb2**	lactamase, beta 2(Lactb2)	6,41E-03
**Lamc2**	laminin, gamma 2(Lamc2)	3,94E-02
**Lats2**	large tumor suppressor 2(Lats2)	3,36E-02
**Lca5**	Leber congenital amaurosis 5 (human)(Lca5)	1,97E-03
**Lcp1**	lymphocyte cytosolic protein 1(Lcp1)	8,36E-04
**Lefty1**	left right determination factor 1(Lefty1)	5,71E-10
**Lefty2**	left-right determination factor 2(Lefty2)	1,02E-02
**Lmbrd2**	LMBR1 domain containing 2(Lmbrd2)	3,85E-03
**Lnpep**	leucyl/cystinyl aminopeptidase(Lnpep)	1,63E-02
**Lpar6**	lysophosphatidic acid receptor 6(Lpar6)	2,76E-03
**Lrp2**	low density lipoprotein receptor-related protein 2(Lrp2)	1,28E-02
**Lrrc46**	leucine rich repeat containing 46(Lrrc46)	4,29E-02
**Lrrtm4**	leucine rich repeat transmembrane neuronal 4(Lrrtm4)	3,09E-04
**Ltf**	lactotransferrin(Ltf)	3,72E-02
**Lyrm7**	LYR motif containing 7(Lyrm7)	5,28E-12
**Lysmd1**	LysM, putative peptidoglycan-binding, domain containing 1(Lysmd1)	1,84E-02
**Lyst**	lysosomal trafficking regulator(Lyst)	3,17E-02
**Man1a**	mannosidase 1, alpha(Man1a)	9,92E-05
**Manba**	mannosidase, beta A, lysosomal(Manba)	4,74E-08
**Map1b**	microtubule-associated protein 1B(Map1b)	1,03E-02
**Mars2**	methionine-tRNA synthetase 2 (mitochondrial)(Mars2)	1,24E-03
**Mast4**	microtubule associated serine/threonine kinase family member 4(Mast4)	2,89E-02
**Mcm2**	minichromosome maintenance complex component 2(Mcm2)	1,98E-02
**Mcm3**	minichromosome maintenance complex component 3(Mcm3)	3,84E-02
**Mcm5**	minichromosome maintenance complex component 5(Mcm5)	9,38E-03
**Med13**	mediator complex subunit 13(Med13)	3,45E-02
**Megf6**	multiple EGF-like-domains 6(Megf6)	2,58E-02
**Met**	met proto-oncogene(Met)	3,48E-02
**Mex3b**	mex3 RNA binding family member B(Mex3b)	3,55E-02
**Mfn2**	mitofusin 2(Mfn2)	1,57E-06
**Micu2**	mitochondrial calcium uptake 2(Micu2)	7,18E-04
**Mob1b**	MOB kinase activator 1B(Mob1b)	2,45E-02
**Mplkip**	M-phase specific PLK1 intereacting protein(Mplkip)	4,20E-02
**Mthfd2**	methylenetetrahydrofolate dehydrogenase (NAD+ dependent), methenyltetrahydrofolate cyclohydrolase(Mthfd2)	6,61E-06
**Mtus2**	microtubule associated tumor suppressor candidate 2(Mtus2)	1,49E-02
**Musk**	muscle, skeletal, receptor tyrosine kinase(Musk)	3,02E-06
**Myb**	myeloblastosis oncogene(Myb)	3,99E-02
**Mylk3**	myosin light chain kinase 3(Mylk3)	6,22E-03
**Myt1l**	myelin transcription factor 1-like(Myt1l)	7,64E-05
**Mzt1**	mitotic spindle organizing protein 1(Mzt1)	1,30E-03
**Nars**	asparaginyl-tRNA synthetase(Nars)	1,78E-08
**Ncapd2**	non-SMC condensin I complex, subunit D2(Ncapd2)	1,73E-02
**Ncf2**	neutrophil cytosolic factor 2(Ncf2)	7,67E-04
**Ndst4**	N-deacetylase/N-sulfotransferase (heparin glucosaminyl) 4(Ndst4)	4,37E-03
**Nek10**	NIMA (never in mitosis gene a)- related kinase 10(Nek10)	1,44E-02
**Nf1**	neurofibromatosis 1(Nf1)	1,71E-04
**Nfe2l3**	nuclear factor, erythroid derived 2, like 3(Nfe2l3)	2,25E-02
**Ngp**	neutrophilic granule protein(Ngp)	4,41E-02
**Nhsl2**	NHS-like 2(Nhsl2)	3,28E-02
**Nkain3**	Na+/K+ transporting ATPase interacting 3(Nkain3)	3,77E-02
**Nodal**	nodal(Nodal)	3,03E-02
**Nos1ap**	nitric oxide synthase 1 (neuronal) adaptor protein(Nos1ap)	1,20E-05
**Notch1**	notch 1(Notch1)	1,90E-03
**Npy2r**	neuropeptide Y receptor Y2(Npy2r)	1,35E-05
**Nr1d1**	nuclear receptor subfamily 1, group D, member 1(Nr1d1)	3,69E-02
**Nr1h4**	nuclear receptor subfamily 1, group H, member 4(Nr1h4)	1,21E-02

Additional File 5: Table S1

**Nr3c2**	nuclear receptor subfamily 3, group C, member 2(Nr3c2)	3,68E-02
**Nrip1**	nuclear receptor interacting protein 1(Nrip1)	3,50E-03
**Nrxn1**	neurexin I(Nrxn1)	9,65E-03
**Nt5c1a**	5'-nucleotidase, cytosolic IA(Nt5c1a)	3,10E-04
**Ntrk3**	neurotrophic tyrosine kinase, receptor, type 3(Ntrk3)	7,33E-03
**Nudcd1**	NudC domain containing 1(Nudcd1)	2,64E-02
**Nudt15**	nudix (nucleoside diphosphate linked moiety X)-type motif 15(Nudt15)	1,41E-02
**Nudt6**	nudix (nucleoside diphosphate linked moiety X)-type motif 6(Nudt6)	2,96E-02
**Nup93**	nucleoporin 93(Nup93)	1,87E-03
**Nvl**	nuclear VCP-like(Nvl)	1,78E-07
**Nyap2**	neuronal tyrosine-phophorylated phosphoinositide 3-kinase adaptor 2(Nyap2)	3,32E-03
**Ogg1**	8-oxoguanine DNA-glycosylase 1(Ogg1)	1,25E-02
**Olfm4**	olfactomedin 4(Olfm4)	2,13E-03
**Olfml3**	olfactomedin-like 3(Olfml3)	2,08E-04
**Olfr316**	olfactory receptor 316(Olfr316)	1,63E-05
**Olfr317**	olfactory receptor 317(Olfr317)	1,47E-02
**Osgep**	O-sialoglycoprotein endopeptidase(Osgep)	3,50E-03
**Palmd**	palmdelphin(Palmd)	2,54E-04
**Paqr8**	progestin and adipoQ receptor family member VIII(Paqr8)	1,41E-05
**Parp1**	poly (ADP-ribose) polymerase family, member 1(Parp1)	1,67E-04
**Pcdh17**	protocadherin 17(Pcdh17)	1,69E-08
**Pcdh9**	protocadherin 9(Pcdh9)	1,87E-04
**Pcdhb12**	protocadherin beta 12(Pcdhb12)	6,27E-06
**Pcdhb15**	protocadherin beta 15(Pcdhb15)	2,18E-05
**Pcdhb16**	protocadherin beta 16(Pcdhb16)	4,60E-04
**Pcdhb17**	protocadherin beta 17(Pcdhb17)	7,96E-04
**Pcdhb18**	protocadherin beta 18(Pcdhb18)	2,87E-03
**Pcdhb19**	protocadherin beta 19(Pcdhb19)	4,22E-03
**Pcdhb9**	protocadherin beta 9(Pcdhb9)	4,74E-08
**Pcdhga10**	protocadherin gamma subfamily A, 10(Pcdhga10)	1,06E-03
**Pcdhga11**	protocadherin gamma subfamily A, 11(Pcdhga11)	7,85E-07
**Pcdhga3**	protocadherin gamma subfamily A, 3(Pcdhga3)	3,18E-03
**Pcdhga8**	protocadherin gamma subfamily A, 8(Pcdhga8)	9,67E-04
**Pcdhga9**	protocadherin gamma subfamily A, 9(Pcdhga9)	9,89E-07
**Pcdhgb1**	protocadherin gamma subfamily B, 1(Pcdhgb1)	2,27E-02
**Pctp**	phosphatidylcholine transfer protein(Pctp)	1,09E-03
**Pde6h**	phosphodiesterase 6H, cGMP-specific, cone, gamma(Pde6h)	4,80E-04
**Pdf**	peptide deformylase (mitochondrial)(Pdf)	3,95E-02
**Pdp2**	pyruvate dehyrogenase phosphatase catalytic subunit 2(Pdp2)	9,48E-03
**Pex5l**	peroxisomal biogenesis factor 5-like(Pex5l)	7,00E-04
**Pi15**	peptidase inhibitor 15(Pi15)	1,14E-02
**Pigw**	phosphatidylinositol glycan anchor biosynthesis, class W(Pigw)	4,89E-02
**Pkd2**	polycystic kidney disease 2(Pkd2)	9,37E-05
**Pkd2l2**	polycystic kidney disease 2-like 2(Pkd2l2)	4,27E-03
**Pkdrej**	polycystin (PKD) family receptor for egg jelly(Pkdrej)	1,49E-02
**Pm20d2**	peptidase M20 domain containing 2(Pm20d2)	1,97E-03
**Pop1**	processing of precursor 1, ribonuclease P/MRP family, (S. cerevisiae)(Pop1)	7,94E-04
**Ppm1e**	protein phosphatase 1E (PP2C domain containing)(Ppm1e)	1,63E-03
**Ppp1r3e**	protein phosphatase 1, regulatory (inhibitor) subunit 3E(Ppp1r3e)	8,07E-08
**Prdm16**	PR domain containing 16(Prdm16)	2,04E-03
**Prickle2**	prickle planar cell polarity protein 2(Prickle2)	4,01E-04
**Prnp**	prion protein(Prnp)	8,64E-07
**Prox1**	prospero homeobox 1(Prox1)	5,80E-03
**Psmb5**	proteasome (prosome, macropain) subunit, beta type 5(Psmb5)	4,98E-04
**Ptchd1**	patched domain containing 1(Ptchd1)	3,27E-02
**Ptchd4**	patched domain containing 4(Ptchd4)	1,58E-04
**Ptgs2**	prostaglandin-endoperoxide synthase 2(Ptgs2)	2,91E-09
**Ptprj**	protein tyrosine phosphatase, receptor type, J(Ptprj)	4,33E-05
**Pttg1**	pituitary tumor-transforming gene 1(Pttg1)	1,45E-02
**Pura**	purine rich element binding protein A(Pura)	8,60E-03
**Rad1**	RAD1 checkpoint DNA exonuclease(Rad1)	9,77E-03
**Rad51ap2**	RAD51 associated protein 2(Rad51ap2)	1,07E-02
**Rasal2**	RAS protein activator like 2(Rasal2)	1,76E-06
**Rbm8a**	RNA binding motif protein 8a(Rbm8a)	1,95E-02
**Rc3h1**	RING CCCH (C3H) domains 1(Rc3h1)	2,45E-02
**Rc3h2**	ring finger and CCCH-type zinc finger domains 2(Rc3h2)	1,08E-02
**Retnlg**	resistin like gamma(Retnlg)	2,84E-02
**Rev3l**	REV3 like, DNA directed polymerase zeta catalytic subunit(Rev3l)	6,26E-05
**Riiad1**	regulatory subunit of type II PKA R-subunit (RIIa) domain containing 1(Riiad1)	2,05E-03
**Rimbp3**	RIMS binding protein 3(Rimbp3)	3,89E-03
**Ror1**	receptor tyrosine kinase-like orphan receptor 1(Ror1)	1,72E-03
**Rorc**	RAR-related orphan receptor gamma(Rorc)	1,36E-05
**Rpl26**	ribosomal protein L26(Rpl26)	7,63E-50
**Rpl34**	ribosomal protein L34(Rpl34)	6,91E-71
**Rpl5**	ribosomal protein L5(Rpl5)	4,18E-08
**Rps19**	ribosomal protein S19(Rps19)	3,20E-02
**Rsg1**	REM2 and RAB-like small GTPase 1(Rsg1)	2,41E-02
**Rsl1**	regulator of sex limited protein 1(Rsl1)	7,44E-03
**S1pr5**	sphingosine-1-phosphate receptor 5(S1pr5)	4,54E-02

Additional File 5: Table S1

**Samd3**	sterile alpha motif domain containing 3(Samd3)	1,06E-02
**Samd5**	sterile alpha motif domain containing 5(Samd5)	4,90E-02
**Scai**	suppressor of cancer cell invasion(Scai)	4,90E-02
**Scn3a**	sodium channel, voltage-gated, type III, alpha(Scn3a)	2,32E-05
**Scnm1**	sodium channel modifier 1(Scnm1)	3,18E-06
**Scoc**	short coiled-coil protein(Scoc)	4,06E-02
**Sec24d**	Sec24 related gene family, member D (S. cerevisiae)(Sec24d)	5,47E-13
**Sema3e**	sema domain, immunoglobulin domain (Ig), short basic domain, secreted, (semaphorin) 3E(Sema3e)	4,90E-03
**Sema5a**	sema domain, seven thrombospondin repeats (type 1 and type 1-like), semaphorin 5A	2,84E-02
**Shroom4**	shroom family member 4(Shroom4)	9,40E-03
**Siglece**	sialic acid binding Ig-like lectin E(Siglece)	5,18E-03
**Sil1**	endoplasmic reticulum chaperone SIL1 homolog (S. cerevisiae)(Sil1)	7,81E-03
**Skil**	SKI-like(Skil)	2,79E-04
**Slc1a5**	solute carrier family 1 (neutral amino acid transporter), member 5(Slc1a5)	3,63E-02
**Slc22a15**	solute carrier family 22 (organic anion/cation transporter), member 15(Slc22a15)	1,94E-04
**Slc2a13**	solute carrier family 2 (facilitated glucose transporter), member 13(Slc2a13)	3,46E-03
**Slc35a5**	solute carrier family 35, member A5(Slc35a5)	1,85E-06
**Slc6a19**	solute carrier family 6 (neurotransmitter transporter), member 19(Slc6a19)	3,64E-02
**Slc7a1**	solute carrier family 7 (cationic amino acid transporter, y+ system), member 1(Slc7a1)	5,99E-05
**Slc7a5**	solute carrier family 7 (cationic amino acid transporter, y+ system), member 5(Slc7a5)	1,36E-05
**Slc8a1**	solute carrier family 8 (sodium/calcium exchanger), member 1(Slc8a1)	1,01E-06
**Slitrk4**	SLIT and NTRK-like family, member 4(Slitrk4)	2,62E-03
**Snrpd1**	small nuclear ribonucleoprotein D1(Snrpd1)	1,67E-06
**Sntn**	sentan, cilia apical structure protein(Sntn)	4,04E-02
**Sorcs3**	sortilin-related VPS10 domain containing receptor 3(Sorcs3)	3,05E-03
**Sorl1**	sortilin-related receptor, LDLR class A repeats-containing(Sorl1)	6,13E-04
**Sox9**	SRY (sex determining region Y)-box 9(Sox9)	3,94E-03
**Sp8**	trans-acting transcription factor 8(Sp8)	4,40E-02
**Srl**	sarcalumenin(Srl)	9,74E-03
**Srp9**	signal recognition particle 9(Srp9)	1,05E-19
**St8sia1**	ST8 alpha-N-acetyl-neuraminide alpha-2,8-sialyltransferase 1(St8sia1)	3,77E-04
**Stab1**	stabilin 1(Stab1)	3,01E-03
**Stac**	src homology three (SH3) and cysteine rich domain(Stac)	2,30E-02
**Stat5a**	signal transducer and activator of transcription 5A(Stat5a)	5,71E-03
**Stk26**	serine/threonine kinase 26(Stk26)	4,37E-02
**Stpg1**	sperm tail PG rich repeat containing 1(Stpg1)	1,06E-02
**Stxbp5l**	syntaxin binding protein 5-like(Stxbp5l)	3,17E-04
**Sult1c1**	sulfotransferase family, cytosolic, 1C, member 1(Sult1c1)	5,34E-03
**Syn3**	synapsin III(Syn3)	5,59E-06
**Syncrip**	synaptotagmin binding, cytoplasmic RNA interacting protein(Syncrip)	4,41E-02
**Syne1**	spectrin repeat containing, nuclear envelope 1(Syne1)	2,05E-02
**Syne2**	spectrin repeat containing, nuclear envelope 2(Syne2)	1,34E-02
**Tanc1**	tetratricopeptide repeat, ankyrin repeat and coiled-coil containing 1(Tanc1)	3,12E-03
**Tbc1d8b**	TBC1 domain family, member 8B(Tbc1d8b)	3,63E-02
**Tchh**	trichohyalin(Tchh)	4,34E-02
**Tdg**	thymine DNA glycosylase(Tdg)	1,15E-04
**Tecta**	tectorin alpha(Tecta)	2,23E-02
**Tenm1**	teneurin transmembrane protein 1(Tenm1)	1,93E-02
**Tenm3**	teneurin transmembrane protein 3(Tenm3)	1,63E-02
**Them4**	thioesterase superfamily member 4(Them4)	2,02E-03
**Thoc2**	THO complex 2(Thoc2)	4,85E-03
**Thrsp**	thyroid hormone responsive(Thrsp)	1,08E-02
**Tll1**	tolloid-like(Tll1)	2,37E-03
**Tmem182**	transmembrane protein 182(Tmem182)	2,34E-02
**Tmem245**	transmembrane protein 245(Tmem245)	3,63E-02
**Tmem260**	transmembrane protein 260(Tmem260)	5,06E-04
**Trib3**	tribbles pseudokinase 3(Trib3)	2,15E-02
**Trim17**	tripartite motif-containing 17(Trim17)	1,07E-03
**Trim2**	tripartite motif-containing 2(Trim2)	2,79E-05
**Trpc4**	transient receptor potential cation channel, subfamily C, member 4(Trpc4)	1,71E-02
**Trpc5**	transient receptor potential cation channel, subfamily C, member 5(Trpc5)	6,11E-04
**Trrap**	transformation/transcription domain-associated protein(Trrap)	8,34E-03
**Ttc30b**	tetratricopeptide repeat domain 30B(Ttc30b)	1,18E-02
**Ttf2**	transcription termination factor, RNA polymerase II(Ttf2)	9,94E-12
**Ttn**	titin(Ttn)	7,74E-03
**Uba1y**	ubiquitin-activating enzyme, Chr Y(Uba1y)	2,21E-02
**Ubxn10**	UBX domain protein 10(Ubxn10)	2,84E-02
**Uhrf1bp1l**	UHRF1 (ICBP90) binding protein 1-like(Uhrf1bp1l)	3,80E-05
**Usp1**	ubiquitin specific peptidase 1(Usp1)	1,72E-04
**Usp2**	ubiquitin specific peptidase 2(Usp2)	5,70E-03
**Usp29**	ubiquitin specific peptidase 29(Usp29)	1,53E-02
**Usp34**	ubiquitin specific peptidase 34(Usp34)	7,00E-06
**Usp53**	ubiquitin specific peptidase 53(Usp53)	1,82E-04
**Vps13c**	vacuolar protein sorting 13C(Vps13c)	1,52E-02
**Vps50**	VPS50 EARP/GARPII complex subunit(Vps50)	2,06E-06
**Vwa3b**	von Willebrand factor A domain containing 3B(Vwa3b)	7,10E-03
**Wdhd1**	WD repeat and HMG-box DNA binding protein 1(Wdhd1)	2,51E-07
**Wipf3**	WAS/WASL interacting protein family, member 3(Wipf3)	3,59E-02
**Xrn1**	5'-3' exoribonuclease 1(Xrn1)	6,22E-05
