# Supplementary material for: Analysis of co-isogenic prion protein deficient mice reveals behavioral deficits, learning impairment, and enhanced hippocampal excitability
Source: BMC Biol. 2022 Jan 13;20:17. doi: 10.1186/s12915-021-01203-0 (PMC8759182; doi:10.1186/s12915-021-01203-0)
Supplement: Supplementary file 6 — Additional file 6: Table S2. List of the protein-coding significantly upregulated genes in PrnpZH3/ZH3 hippocampus compared to Prnp+/+. [file 12915_2021_1203_MOESM6_ESM.pdf]

Additional File 6: Table S2

Upregulated genes in *Prnp* $^{ZH3/ZH3}$ compared to *Prnp* $^{+/+}$

Gene	Name	padj_Ko vs Wt
Abhd8	abhydrolase domain containing 8(Abhd8)	0,000108971
Acad12	acyl-Coenzyme A dehydrogenase family, member 12(Acad12)	0,00370349
Adamts2	a disintegrin-like and metallopeptidase (reprolysin type) with thrombospondin type 1 motif, 2(Adamts2)	0,013525708
Adamtsl5	ADAMTS-like 5(Adamtsl5)	2,82101E-05
Adarb2	adenosine deaminase, RNA-specific, B2(Adarb2)	0,007849456
Adhfe1	alcohol dehydrogenase, iron containing, 1(Adhfe1)	0,002190853
Adora2b	adenosine A2b receptor(Adora2b)	4,24189E-18
Adrb2	adrenergic receptor, beta 2(Adrb2)	0,000439986
AI987944	expressed sequence AI987944(AI987944)	1,92487E-14
Ajuba	ajuba LIM protein(Ajuba)	0,005071766
Aldh1a3	aldehyde dehydrogenase family 1, subfamily A3(Aldh1a3)	0,000443768
Aldh1l1	aldehyde dehydrogenase 1 family, member L1(Aldh1l1)	0,001718877
Anapc13	anaphase promoting complex subunit 13(Anapc13)	1,99277E-06
Angptl7	angiopoietin-like 7(Angptl7)	0,024025842
Apoc3	apolipoprotein C-III(Apoc3)	0,019261329
Arhgap26	Rho GTPase activating protein 26(Arhgap26)	1,09315E-08
Arhgap33	Rho GTPase activating protein 33(Arhgap33)	0,000123622
Arhgdig	Rho GDP dissociation inhibitor (GDI) gamma(Arhgdig)	3,15408E-07
Arl5c	ADP-ribosylation factor-like 5C(Arl5c)	0,026903754
Aspdh	aspartate dehydrogenase domain containing(Aspdh)	0,017047962
Aspg	asparaginase(Aspg)	0,006771728
Atp5d	ATP synthase, H+ transporting, mitochondrial F1 complex, delta subunit(Atp5d)	0,00039469
Atp5e	ATP synthase, H+ transporting, mitochondrial F1 complex, epsilon subunit(Atp5e)	2,71606E-05
Atp5j2	ATP synthase, H+ transporting, mitochondrial F0 complex, subunit F2(Atp5j2)	0,000951904
B3gnt7	UDP-GlcNAc:betaGal beta-1,3-N-acetylglucosaminyltransferase 7(B3gnt7)	0,026262529
B9d1	B9 protein domain 1(B9d1)	0,00013909
Bace2	beta-site APP-cleaving enzyme 2(Bace2)	0,04258515
Baiap2l1	BAI1-associated protein 2-like 1(Baiap2l1)	3,46451E-05
Blnk	B cell linker(Blnk)	0,01909235
Blvrb	biliverdin reductase B (flavin reductase (NADPH))(Blvrb)	0,003374943
Bola2	bolA-like 2 (E. coli)(Bola2)	0,001621668
C1qa	complement component 1, q subcomponent, alpha polypeptide(C1qa)	0,001061274
C2cd4c	C2 calcium-dependent domain containing 4C(C2cd4c)	0,003273898
C77080	expressed sequence C77080(C77080)	0,001282304
Car8	carbonic anhydrase 8(Car8)	0,001173458
Cartpt	CART prepropeptide(Cartpt)	0,037712862
Casq2	calsequestrin 2(Casq2)	0,00014535
Catsperg2	catsper channel auxiliary subunit gamma 2(Catsperg2)	1,90518E-10
Cav3	caveolin 3(Cav3)	0,041711219
Ccnb1ip1	cyclin B1 interacting protein 1(Ccnb1ip1)	0,003848558
Cd14	CD14 antigen(Cd14)	0,000374514
Cd209b	CD209b antigen(Cd209b)	0,042559733
Cd209g	CD209g antigen(Cd209g)	0,042785929
Cd244	CD244 natural killer cell receptor 2B4(Cd244)	0,013998529
Cdh23	cadherin 23 (otocadherin)(Cdh23)	0,044266118
Cdh24	cadherin-like 24(Cdh24)	2,29496E-33
Ceacam15	carcinoembryonic antigen-related cell adhesion molecule 15(Ceacam15)	0,022321421
Cebpa	CCAAT/enhancer binding protein (C/EBP), alpha(Cebpa)	0,018975288
Cgref1	cell growth regulator with EF hand domain 1(Cgref1)	0,026222006
Cib2	calcium and integrin binding family member 2(Cib2)	2,7864E-05
Clcnka	chloride channel, voltage-sensitive Ka(Clcnka)	0,0009127
Cldn10	claudin 10(Cldn10)	3,44589E-10
Clec11a	C-type lectin domain family 11, member a(Clec11a)	0,025691417
Cmbl	carboxymethylenebutenolidase-like (Pseudomonas)(Cmbl)	0,04339448
Col11a1	collagen, type XI, alpha 1(Col11a1)	0,020643276
Col16a1	collagen, type XVI, alpha 1(Col16a1)	0,023948264
Col9a2	collagen, type IX, alpha 2(Col9a2)	0,009065738
Colgalt2	collagen beta(1-O)galactosyltransferase 2(Colgalt2)	1,89405E-12
Coq8b	coenzyme Q8B(Coq8b)	4,28282E-05
Cox14	cytochrome c oxidase assembly protein 14(Cox14)	0,000252078
Cox16	cytochrome c oxidase assembly protein 16(Cox16)	0,02205668
Cox18	cytochrome c oxidase assembly protein 18(Cox18)	0,010310937
Cox8a	cytochrome c oxidase subunit VIIIa(Cox8a)	0,000273249
Cplx1	complexin 1(Cplx1)	7,7592E-12
Crhr1	corticotropin releasing hormone receptor 1(Crhr1)	0,0212643
Csf3r	colony stimulating factor 3 receptor (granulocyte)(Csf3r)	0,014773473
Cst6	cystatin E/M(Cst6)	0,045690563
Ctu1	cytosolic thiouridylase subunit 1(Ctu1)	4,94178E-05
Cyp2j13	cytochrome P450, family 2, subfamily j, polypeptide 13(Cyp2j13)	9,64977E-05
Cyp2j9	cytochrome P450, family 2, subfamily j, polypeptide 9(Cyp2j9)	3,6628E-07
Cyp4f14	cytochrome P450, family 4, subfamily f, polypeptide 14(Cyp4f14)	3,50098E-05
Cyr61	cysteine rich protein 61(Cyr61)	0,003444989
D8Ertd738e	DNA segment, Chr 8, ERATO Doi 738, expressed(D8Ertd738e)	2,54235E-06
Dapl1	death associated protein-like 1(Dapl1)	0,034745775

Additional File 6: Table S2

**Derl3**	Der1-like domain family, member 3(Derl3)	0,028116083
**Dgcr6**	DiGeorge syndrome critical region gene 6(Dgcr6)	0,000398309
**Dhrs3**	dehydrogenase/reductase (SDR family) member 3(Dhrs3)	1,24633E-06
**Diaph3**	diaphanous related formin 3(Diaph3)	0,011686966
**Dmpk**	dystrophia myotonica-protein kinase(Dmpk)	0,004617504
**Dnah14**	dynein, axonemal, heavy chain 14(Dnah14)	5,02964E-15
**Draxin**	dorsal inhibitory axon guidance protein(Draxin)	0,011359434
**Emc9**	ER membrane protein complex subunit 9(Emc9)	0,006178541
**Eno1b**	enolase 1B, retrotransposed(Eno1b)	1,66114E-17
**Epb41l4a**	erythrocyte membrane protein band 4.1 like 4a(Epb41l4a)	0,005523429
**Ephx2**	epoxide hydrolase 2, cytoplasmic(Ephx2)	4,05297E-05
**Ethe1**	ethylmalonic encephalopathy 1(Ethe1)	0,005413669
**Fahd2a**	fumarylacetoacetate hydrolase domain containing 2A(Fahd2a)	0,001626607
**Fars2**	phenylalanine-tRNA synthetase 2 (mitochondrial)(Fars2)	0,000286633
**Fau**	Finkel-Biskis-Reilly murine sarcoma virus (FBR-MuSV) ubiquitously expressed (fox derived)(Fau)	7,54284E-05
**Fbxo2**	F-box protein 2(Fbxo2)	5,79723E-06
**Fbxo6**	F-box protein 6(Fbxo6)	3,13747E-06
**Fcgr1**	Fc receptor, IgG, high affinity I(Fcgr1)	0,001805163
**Fchsd1**	FCH and double SH3 domains 1(Fchsd1)	0,028000124
**Fgd2**	FYVE, RhoGEF and PH domain containing 2(Fgd2)	0,007298807
**Filip1l**	filamin A interacting protein 1-like(Filip1l)	0,001506306
**Foxo6**	forkhead box O6(Foxo6)	0,040219376
**Frmd3**	FERM domain containing 3(Frmd3)	0,02462044
**Fxyd1**	FXYD domain-containing ion transport regulator 1(Fxyd1)	0,048938601
**Fxyd6**	FXYD domain-containing ion transport regulator 6(Fxyd6)	0,04378888
**Fzd1**	frizzled class receptor 1(Fzd1)	0,048910076
**Gabra2**	gamma-aminobutyric acid (GABA) A receptor, subunit alpha 2(Gabra2)	0,002552168
**Gadd45b**	growth arrest and DNA-damage-inducible 45 beta(Gadd45b)	0,020103162
**Gal**	galanin(Gal)	0,045213367
**Gbp2b**	guanylate binding protein 2b(Gbp2b)	1,87894E-07
**Ghsr**	growth hormone secretagogue receptor(Ghsr)	0,000278679
**Gimap6**	GTPase, IMAP family member 6(Gimap6)	0,029150268
**Gipc1**	GIPC PDZ domain containing family, member 1(Gipc1)	2,38641E-05
**Gng13**	guanine nucleotide binding protein (G protein), gamma 13(Gng13)	5,02712E-09
**Gon4l**	gon-4-like (C.elegans)(Gon4l)	8,53386E-13
**Gpat2**	glycerol-3-phosphate acyltransferase 2, mitochondrial(Gpat2)	0,005660112
**Gpatch11**	G patch domain containing 11(Gpatch11)	0,000160568
**Gpld1**	glycosylphosphatidylinositol specific phospholipase D1(Gpld1)	0,000730313
**Gpx3**	glutathione peroxidase 3(Gpx3)	0,009403913
**Grap**	GRB2-related adaptor protein(Grap)	0,000110831
**Grem2**	gremlin 2, DAN family BMP antagonist(Grem2)	1,27291E-05
**Grk4**	G protein-coupled receptor kinase 4(Grk4)	0,018519885
**Gsta4**	glutathione S-transferase, alpha 4(Gsta4)	7,05251E-07
**Gstp1**	glutathione S-transferase, pi 1(Gstp1)	0,004849194
**Gucy2c**	guanylate cyclase 2c(Gucy2c)	0,008990065
**H2-Q2**	histocompatibility 2, Q region locus 2(H2-Q2)	0,047318323
**H2-T23**	histocompatibility 2, T region locus 23(H2-T23)	0,044740374
**H2-T24**	histocompatibility 2, T region locus 24(H2-T24)	0,036180825
**Hacd1**	3-hydroxyacyl-CoA dehydratase 1(Hacd1)	0,003737597
**Hap1**	huntingtin-associated protein 1(Hap1)	0,009139401
**Haus4**	HAUS augmin-like complex, subunit 4(Haus4)	6,24157E-08
**Hddc3**	HD domain containing 3(Hddc3)	0,002322164
**Hebp2**	heme binding protein 2(Hebp2)	0,014896141
**Hist1h2ba**	histone cluster 1, H2ba(Hist1h2ba)	0,00024879
**Hist2h2bb**	histone cluster 2, H2bb(Hist2h2bb)	1,17483E-12
**Hmgcs2**	3-hydroxy-3-methylglutaryl-Coenzyme A synthase 2(Hmgcs2)	0,002194486
**Hn1**	hematological and neurological expressed sequence 1(Hn1)	3,54789E-07
**Hnf1b**	HNF1 homeobox B(Hnf1b)	0,000123622
**Hpn**	hepsin(Hpn)	0,016575122
**Hrh2**	histamine receptor H2(Hrh2)	0,01214204
**Hspb7**	heat shock protein family, member 7 (cardiovascular)(Hspb7)	0,039219681
**Htra3**	HtrA serine peptidase 3(Htra3)	0,047713851
**Ifi202b**	interferon activated gene 202B(Ifi202b)	5,78911E-09
**Igfbp3**	insulin-like growth factor binding protein 3(Igfbp3)	0,010686063
**Igfbp6**	insulin-like growth factor binding protein 6(Igfbp6)	0,006690813
**Igsf21**	immunoglobulin superfamily, member 21(Igsf21)	0,001969598
**Il10ra**	interleukin 10 receptor, alpha(Il10ra)	0,000138811
**Inpp4a**	inositol polyphosphate-4-phosphatase, type I(Inpp4a)	4,28282E-05
**Invs**	inversin(Invs)	0,003682237
**Irf5**	interferon regulatory factor 5(Irf5)	0,006063789
**Itih3**	inter-alpha trypsin inhibitor, heavy chain 3(Itih3)	8,19695E-05
**Kcne1l**	potassium voltage-gated channel, Isk-related family, member 1-like, pseudogene(Kcne1l)	0,035393066
**Kcnh6**	potassium voltage-gated channel, subfamily H (eag-related), member 6(Kcnh6)	0,003501711
**Kcnj16**	potassium inwardly-rectifying channel, subfamily J, member 16(Kcnj16)	0,000245359
**Klhl33**	kelch-like 33(Klhl33)	0,023876645
**Klrg1**	killer cell lectin-like receptor subfamily G, member 1(Klrg1)	0,000290116
**Kptn**	kaptin(Kptn)	4,48332E-11
**Lcat**	lecithin cholesterol acyltransferase(Lcat)	0,00115684
**Ldhd**	lactate dehydrogenase D(Ldhd)	8,09733E-09

Additional File 6: Table S2

**Lgi4**	leucine-rich repeat LGI family, member 4(Lgi4)	9,93698E-12
**Limk1**	LIM-domain containing, protein kinase(Limk1)	0,000431861
**Lims2**	LIM and senescent cell antigen like domains 2(Lims2)	0,029705811
**Lpl**	lipoprotein lipase(Lpl)	0,024385469
**Lrrc27**	leucine rich repeat containing 27(Lrrc27)	0,000137807
**Lsm3**	LSM3 homolog, U6 small nuclear RNA and mRNA degradation associated(Lsm3)	0,001412329
**Lsp1**	lymphocyte specific 1(Lsp1)	0,000236691
**Lst1**	leukocyte specific transcript 1(Lst1)	0,003984899
**Ly6h**	lymphocyte antigen 6 complex, locus H(Ly6h)	0,033625344
**Ly86**	lymphocyte antigen 86(Ly86)	0,016535425
**Lyplal1**	lysophospholipase-like 1(Lyplal1)	1,0244E-14
**Mad2l2**	MAD2 mitotic arrest deficient-like 2(Mad2l2)	7,67117E-06
**Mael**	maelstrom spermatogenic transposon silencer(Mael)	0,035363452
**March3**	membrane-associated ring finger (C3HC4) 3(March3)	0,045087706
**Mdk**	midkine(Mdk)	0,014512013
**Mien1**	migration and invasion enhancer 1(Mien1)	3,30779E-10
**Mill2**	MHC I like leukocyte 2(Mill2)	0,000506448
**Mme**	membrane metallo endopeptidase(Mme)	0,001023525
**Mrap2**	melanocortin 2 receptor accessory protein 2(Mrap2)	0,005085478
**Mroh5**	maestro heat-like repeat family member 5(Mroh5)	0,034391061
**Mroh7**	maestro heat-like repeat family member 7(Mroh7)	0,010174898
**Mrpl34**	mitochondrial ribosomal protein L34(Mrpl34)	0,029340219
**Mt3**	metallothionein 3(Mt3)	7,87719E-06
**Mttp**	microsomal triglyceride transfer protein(Mttp)	1,09827E-08
**Mup5**	major urinary protein 5(Mup5)	0,020290342
**Mxra7**	matrix-remodelling associated 7(Mxra7)	0,009825154
**Myh6**	myosin, heavy polypeptide 6, cardiac muscle, alpha(Myh6)	2,20426E-13
**Myh8**	myosin, heavy polypeptide 8, skeletal muscle, perinatal(Myh8)	2,90074E-09
**Myl1**	myosin, light polypeptide 1(Myl1)	0,002327962
**Nat8f5**	N-acetyltransferase 8 (GCN5-related) family member 5(Nat8f5)	0,023403006
**Ndn**	necdin(Ndn)	8,76444E-08
**Ndrg2**	N-myc downstream regulated gene 2(Ndrg2)	1,20517E-15
**Nedd8**	neural precursor cell expressed, developmentally down-regulated gene 8(Nedd8)	1,03678E-05
**Ngdn**	neuroguidin, EIF4E binding protein(Ngdn)	4,42626E-06
**Nkain4**	Na+/K+ transporting ATPase interacting 4(Nkain4)	2,32098E-05
**Nnat**	neuronatin(Nnat)	0,032092937
**Npas1**	neuronal PAS domain protein 1(Npas1)	0,003227579
**Nr2f6**	nuclear receptor subfamily 2, group F, member 6(Nr2f6)	0,02166182
**Nrk**	Nik related kinase(Nrk)	0,047417636
**Nrsn1**	neurensin 1(Nrsn1)	5,15932E-08
**Ntn4**	netrin 4(Ntn4)	8,98546E-05
**Nudt2**	nudix (nucleoside diphosphate linked moiety X)-type motif 2(Nudt2)	0,000620555
**Ost4**	oligosaccharyltransferase complex subunit 4 (non-catalytic)(Ost4)	9,38489E-05
**Ostf1**	osteoclast stimulating factor 1(Ostf1)	0,00035024
**Oxtr**	oxytocin receptor(Oxtr)	0,032103073
**P4ha3**	procollagen-proline, 2-oxoglutarate 4-dioxygenase (proline 4-hydroxylase), alpha polypeptide III(P4ha3)	0,015854643
**Padi2**	peptidyl arginine deiminase, type II(Padi2)	0,007437629
**Palm3**	paralemmin 3(Palm3)	0,006189613
**Papss2**	3'-phosphoadenosine 5'-phosphosulfate synthase 2(Papss2)	0,005787804
**Pcbd2**	pterin 4 alpha carbinolamine dehydratase/dimerization cofactor of hepatocyte nuclear factor 1 alpha (TCF1) 2(Pcbd2)	0,007332987
**Pcdhb3**	protocadherin beta 3(Pcdhb3)	8,19695E-05
**Pcdhb6**	protocadherin beta 6(Pcdhb6)	0,001327989
**Pcdhb7**	protocadherin beta 7(Pcdhb7)	0,000432829
**Pcdhb8**	protocadherin beta 8(Pcdhb8)	6,62307E-06
**Pcsk1n**	proprotein convertase subtilisin/kexin type 1 inhibitor(Pcsk1n)	0,008338887
**Pdzk1**	PDZ domain containing 1(Pdzk1)	0,007782851
**Pgbd1**	piggyBac transposable element derived 1(Pgbd1)	0,016680571
**Pigf**	phosphatidylinositol glycan anchor biosynthesis, class F(Pigf)	0,005869219
**Pin1**	protein (peptidyl-prolyl cis/trans isomerase) NIMA-interacting 1(Pin1)	0,005918898
**Pip5kl1**	phosphatidylinositol-4-phosphate 5-kinase-like 1(Pip5kl1)	0,014731124
**Pkp1**	plakophilin 1(Pkp1)	0,019978324
**Plscr2**	phospholipid scramblase 2(Plscr2)	0,014896141
**Plvap**	plasmalemma vesicle associated protein(Plvap)	0,008338887
**Podxl2**	podocalyxin-like 2(Podxl2)	0,020259441
**Ppp1r3g**	protein phosphatase 1, regulatory (inhibitor) subunit 3G(Ppp1r3g)	0,014655538
**Proca1**	protein interacting with cyclin A1(Proca1)	5,37244E-08
**Proser3**	proline and serine rich 3(Proser3)	0,024183861
**Prtn3**	proteinase 3(Prtn3)	0,045690563
**Psmb10**	proteasome (prosome, macropain) subunit, beta type 10(Psmb10)	4,14628E-05
**Psmb11**	proteasome (prosome, macropain) subunit, beta type, 11(Psmb11)	9,03225E-16
**Psmg4**	proteasome (prosome, macropain) assembly chaperone 4(Psmg4)	0,000308357
**Pth1r**	parathyroid hormone 1 receptor(Pth1r)	0,012803732
**Pvalb**	parvalbumin(Pvalb)	0,009247559
**Pxmp2**	peroxisomal membrane protein 2(Pxmp2)	0,000309658
**Pyroxd2**	pyridine nucleotide-disulphide oxidoreductase domain 2(Pyroxd2)	0,000209316
**Qsox1**	quiescin Q6 sulfhydryl oxidase 1(Qsox1)	3,15507E-07
**Rabac1**	Rab acceptor 1 (prenylated)(Rabac1)	2,13046E-06
**Ramp1**	receptor (calcitonin) activity modifying protein 1(Ramp1)	0,000326559
**Rdh13**	retinol dehydrogenase 13 (all-trans and 9-cis)(Rdh13)	2,45414E-05

Additional File 6: Table S2

**Rdm1**	RAD52 motif 1(Rdm1)	0,00021784
**Reg3b**	regenerating islet-derived 3 beta(Reg3b)	0,000705532
**Resp18**	regulated endocrine-specific protein 18(Resp18)	0,038218746
**Rgcc**	regulator of cell cycle(Rgcc)	0,027545958
**Rgsl1**	regulator of G-protein signaling like 1(Rgsl1)	0,012803732
**Rnase1**	ribonuclease, RNase A family, 1 (pancreatic)(Rnase1)	9,11616E-16
**Rnaseh2c**	ribonuclease H2, subunit C(Rnaseh2c)	0,003719927
**Rnd2**	Rho family GTPase 2(Rnd2)	0,000598745
**Rnf223**	ring finger 223(Rnf223)	0,012886903
**Rnls**	renalase, FAD-dependent amine oxidase(Rnls)	0,030187967
**Romo1**	reactive oxygen species modulator 1(Romo1)	0,002521045
**Rpa2**	replication protein A2(Rpa2)	2,32898E-05
**Rplp1**	ribosomal protein, large, P1(Rplp1)	0,001762362
**Rxfp3**	relaxin family peptide receptor 3(Rxfp3)	0,025262545
**S100b**	S100 protein, beta polypeptide, neural(S100b)	6,62307E-06
**Sag**	S-antigen, retina and pineal gland (arrestin)(Sag)	0,005760837
**Samd11**	sterile alpha motif domain containing 11(Samd11)	0,024128788
**Scrg1**	scrapie responsive gene 1(Scrg1)	0,029641461
**Sdhc**	succinate dehydrogenase complex, subunit C, integral membrane protein(Sdhc)	3,23692E-11
**Selenbp2**	selenium binding protein 2(Selenbp2)	7,60827E-10
**Sema4g**	sema domain, immunoglobulin domain (Ig),(semaphorin) 4G(Sema4g)	0,001488735
**Serf1**	small EDRK-rich factor 1(Serf1)	2,01762E-06
**Serpina3f**	serine (or cysteine) peptidase inhibitor, clade A, member 3F(Serpina3f)	0,006759948
**Serpind1**	serine (or cysteine) peptidase inhibitor, clade D, member 1(Serpind1)	0,002291179
**Sgpp2**	sphingosine-1-phosphate phosphotase 2(Sgpp2)	0,047318323
**Sgsm2**	small G protein signaling modulator 2(Sgsm2)	1,99277E-06
**Sh2b2**	SH2B adaptor protein 2(Sh2b2)	0,005441559
**Shh**	sonic hedgehog(Shh)	0,044428741
**Slamf8**	SLAM family member 8(Slamf8)	5,06196E-07
**Slc13a5**	solute carrier family 13 (sodium-dependent citrate transporter), member 5(Slc13a5)	0,000112413
**Slc15a3**	solute carrier family 15, member 3(Slc15a3)	0,007430117
**Slc16a1**	solute carrier family 16 (monocarboxylic acid transporters), member 1(Slc16a1)	0,020817532
**Slc1a6**	solute carrier family 1 (high affinity aspartate/glutamate transporter), member 6(Slc1a6)	0,030729958
**Slc25a31**	solute carrier family 25 (mitochondrial carrier; adenine nucleotide translocator), member 31(Slc25a31)	0,018115939
**Slc2a5**	solute carrier family 2 (facilitated glucose transporter), member 5(Slc2a5)	0,000676652
**Slc30a2**	solute carrier family 30 (zinc transporter), member 2(Slc30a2)	0,024438813
**Slc36a2**	solute carrier family 36 (proton/amino acid symporter), member 2(Slc36a2)	0,044726931
**Slc7a7**	solute carrier family 7 (cationic amino acid transporter, y+ system), member 7(Slc7a7)	0,04830052
**Smdt1**	single-pass membrane protein with aspartate rich tail 1(Smdt1)	1,26407E-05
**Smim1**	small integral membrane protein 1(Smim1)	0,002788164
**Smyd1**	SET and MYND domain containing 1(Smyd1)	0,001871246
**Sncb**	synuclein, beta(Sncb)	0,004661403
**Snrnp25**	small nuclear ribonucleoprotein 25 (U11/U12)(Snrnp25)	2,60939E-05
**Sp100**	nuclear antigen Sp100(Sp100)	0,033739435
**Sparc**	secreted acidic cysteine rich glycoprotein(Sparc)	0,003889208
**Spink10**	serine peptidase inhibitor, Kazal type 10(Spink10)	0,035313138
**Stk10**	serine/threonine kinase 10(Stk10)	0,019988929
**Strc**	stereocilin(Strc)	0,007385701
**Sumf2**	sulfatase modifying factor 2(Sumf2)	0,003052813
**Susd2**	sushi domain containing 2(Susd2)	0,003052813
**Susd5**	sushi domain containing 5(Susd5)	0,006459583
**Swt1**	SWT1 RNA endoribonuclease homolog (S. cerevisiae)(Swt1)	0,002117681
**Sytl1**	synaptotagmin-like 1(Sytl1)	0,003088137
**Tbc1d9b**	TBC1 domain family, member 9B(Tbc1d9b)	4,33403E-20
**Tcap**	titin-cap(Tcap)	0,000401008
**Tgfb1**	transforming growth factor, beta 1(Tgfb1)	0,007728873
**Timp4**	tissue inhibitor of metalloproteinase 4(Timp4)	9,3705E-06
**Tlcd2**	TLC domain containing 2(Tlcd2)	0,022415558
**Tlr1**	toll-like receptor 1(Tlr1)	0,0128052
**Tlr6**	toll-like receptor 6(Tlr6)	0,022460838
**Tmem132e**	transmembrane protein 132E(Tmem132e)	0,03038241
**Tmem173**	transmembrane protein 173(Tmem173)	0,000334003
**Tmem176a**	transmembrane protein 176A(Tmem176a)	0,038218746
**Tmem30c**	transmembrane protein 30C(Tmem30c)	0,014993972
**Tmem74b**	transmembrane protein 74B(Tmem74b)	0,012898105
**Tmod3**	tropomodulin 3(Tmod3)	0,00275412
**Tmod4**	tropomodulin 4(Tmod4)	0,000122148
**Tmsb10**	thymosin, beta 10(Tmsb10)	0,00167863
**Tnfaip8**	tumor necrosis factor, alpha-induced protein 8(Tnfaip8)	0,000483409
**Tpm2**	tropomyosin 2, beta(Tpm2)	0,007924549
**Trabd2b**	TraB domain containing 2B(Trabd2b)	0,019580787
**Traf3ip3**	TRAF3 interacting protein 3(Traf3ip3)	0,015136822
**Trappc6a**	trafficking protein particle complex 6A(Trappc6a)	0,000178642
**Tsks**	testis-specific serine kinase substrate(Tsks)	0,007782851
**Tspan17**	tetraspanin 17(Tspan17)	8,61665E-05
**Ttc9b**	tetratricopeptide repeat domain 9B(Ttc9b)	0,017345165
**Ttk**	Ttk protein kinase(Ttk)	0,015098252
**Tuba1c**	tubulin, alpha 1C(Tuba1c)	0,0254744
**Tusc1**	tumor suppressor candidate 1(Tusc1)	0,040833696

Additional File 6: Table S2

**Tyrobp**	TYRO protein tyrosine kinase binding protein(Tyrobp)	0,000181857
**Uba5**	ubiquitin-like modifier activating enzyme 5(Uba5)	6,89074E-24
**Unc119**	unc-119 lipid binding chaperone(Unc119)	0,000251962
**Uqcc2**	ubiquinol-cytochrome c reductase complex assembly factor 2(Uqcc2)	0,00010448
**Uqcc3**	ubiquinol-cytochrome c reductase complex assembly factor 3(Uqcc3)	1,79836E-05
**Uqcr11**	ubiquinol-cytochrome c reductase, complex III subunit XI(Uqcr11)	0,000374312
**Uqcrq**	ubiquinol-cytochrome c reductase, complex III subunit VII(Uqcrq)	0,001631318
**Uty**	ubiquitously transcribed tetratricopeptide repeat gene, Y chromosome(Uty)	0,000835085
**Wbscr27**	Williams Beuren syndrome chromosome region 27 (human)(Wbscr27)	0,01110656
**Wdfy1**	WD repeat and FYVE domain containing 1(Wdfy1)	0,000122974
**Wnt4**	wingless-type MMTV integration site family, member 4(Wnt4)	0,009155246
**Wnt7a**	wingless-type MMTV integration site family, member 7A(Wnt7a)	0,01214204
